# Birdsong: Is It Music to Their Ears?

**DOI:** 10.3389/fnevo.2012.00014

**Published:** 2012-11-28

**Authors:** Sarah E. Earp, Donna L. Maney

**Affiliations:** ^1^Department of Psychology, Emory UniversityAtlanta, GA, USA

**Keywords:** Egr-1, mesolimbic reward system, reward, music, song, songbird

## Abstract

Since the time of Darwin, biologists have wondered whether birdsong and music may serve similar purposes or have the same evolutionary precursors. Most attempts to compare song with music have focused on the qualities of the sounds themselves, such as melody and rhythm. Song is a signal, however, and as such its meaning is tied inextricably to the response of the receiver. Imaging studies in humans have revealed that hearing music induces neural responses in the mesolimbic reward pathway. In this study, we tested whether the homologous pathway responds in songbirds exposed to conspecific song. We played male song to laboratory-housed white-throated sparrows, and immunolabeled the immediate early gene product Egr-1 in each region of the reward pathway that has a clear or putative homologue in humans. We found that the responses, and how well they mirrored those of humans listening to music, depended on sex and endocrine state. In females with breeding-typical plasma levels of estradiol, all of the regions of the mesolimbic reward pathway that respond to music in humans responded to song. In males, we saw responses in the amygdala but not the nucleus accumbens – similar to the pattern reported in humans listening to unpleasant music. The shared responses in the evolutionarily ancient mesolimbic reward system suggest that birdsong and music engage the same neuroaffective mechanisms in the intended listeners.

## Introduction

Ornithologists and musicians alike have long contemplated whether the song of birds might somehow be classified as “music.” The question can be approached from a variety of angles, each of which produces a somewhat different answer. Researchers have asked, for example, whether birdsong and music share evolutionary precursors or functions (Darwin, 1871; Catchpole and Slater, 1995; Miller, 2001), tonal variation or rhythm (Dobson and Lemon, 1977; Slater, 2001; Baptista and Keister, 2005; Araya-Salas, 2012), or organization (Marler, 2001), and whether, like music, birdsong is creative (Marler, 2001; Hartshorne, 2008). Whether any particular species of songbird has music-like song depends on the parameter measured and the type of analysis employed.

Birdsong, hereafter referred to as song, is a signal; it has a sender and a receiver. Ultimately, a signal’s effect on the receiver, not its structure, dictates its meaning and function (reviewed by Scott-Phillips, 2008). When comparing song and music, it may therefore be informative to ask about the receiver’s response and subjective experience. Human listeners find music rewarding; they will approach it and work to hear it. Songbirds of many species likewise show a phonotaxic response to conspecific song. Female pied flycatchers (*Ficedula albicollis*) and European starlings (*Sturnus vulgaris*) approach and enter nest boxes containing speakers playing male song (Eriksson and Wallin, 1986; Gentner and Hulse, 2000), and female zebra finches (*Taeniopygia guttata*) will peck a key to hear male song (Riebel, 2000). Young male zebra finches who are learning to sing will also peck to hear song (Adret, 1993), but in general, a phonotaxic effect of song is less pronounced in male songbirds than in females (Dobson and Petrinovich, 1973; Stevenson-Hinde and Roper, 1975).

Measuring behavioral responses is but one way to assess the effects of a signal on the receiver. Over the past decade, neuroimaging studies have identified at least 20 different brain regions that show altered BOLD or PET responses during music listening. Some of the most commonly reported responses, particularly to music that is pleasurable to the listener, are those of the mesolimbic reward system. This system consists of the ventral tegmental area (VTA) and its dopaminergic projections to several regions of the forebrain, for example the nucleus accumbens (nAc) in the ventral striatum. Release of dopamine in nAc occurs at precisely the time that intensely pleasurable autonomic responses, or “chills,” are experienced during music listening (Salimpoor et al., 2011). Although the release itself may not itself *cause* the experience of reward, it indicates that the stimulus is associated with reward (reviewed by Wise, 2004). Also included in the reward system are the dorsal striatum (e.g., caudate nucleus in humans), the heavily interconnected amygdala and hippocampus (Hp), and the prefrontal cortex. Each of these regions have been shown in multiple human imaging studies to respond to music with BOLD or PET responses (Blood and Zatorre, 2001; Koelsch et al., 2006; Mitterschiffthaler et al., 2007; Montag et al., 2011; Pereira et al., 2011; Salimpoor et al., 2011).

In this study we looked for neural responses to song in the avian homologues of music-responsive brain regions. Functional MRI can be used in songbirds listening to song (Van Meir et al., 2005; Boumans et al., 2007), but to date those analyses have focused primarily on the major auditory areas. The nAc and other areas known to respond to music in humans are difficult to study using this technique in songbirds, primarily because of their small size. Neural responses to stimuli can be more readily studied in birds by mapping the expression of immediate early genes (IEGs) such as Fos and Egr-1. In such studies, a stimulus is presented to an animal and the brain harvested 60–90 min later. The protein products of IEGs can then be labeled in fixed brain sections using immunohistochemistry, which provides cellular resolution. Dubbed the “genomic action potential” (Clayton, 2000), the IEG response indicates that a neuron has begun to respond to a stimulus with new protein synthesis related to synaptic remodeling. Although the IEG and BOLD responses make use of different underlying molecular mechanisms, there is good agreement between results obtained by both methods (Lazovic et al., 2005; Stark et al., 2006). In songbirds, for example, hearing song induces robust Egr-1 and BOLD responses in the auditory forebrain (Mello et al., 1992; Gentner et al., 2001; Van Meir et al., 2005; Boumans et al., 2007). Egr-1 is particularly useful in the study of reward because it appears to play an active role in the reward process. In rodents, Egr-1 is induced in the reward pathway by drugs such as methamphetamine, morphine, nicotine, or cocaine (reviewed by Girault et al., 2007). Blockade of Egr-1 prevents conditioned behavioral responses to these drugs, suggesting that Egr-1 not only marks neuronal responses to reward but is required for the acquisition of reward-reinforced behaviors.

In this study, we used Egr-1 as a marker to map and quantify neural responses in the mesolimbic reward system in male and female white-throated sparrows (*Zonotrichia albicollis*) listening to conspecific male song. This species sings a particularly musical-sounding song (Saunders, 1959) with heavy use of whistles with a sustained pitch (Dobson and Lemon, 1977). During the non-breeding season, song is used by both sexes to establish and maintain dominance relationships (reviewed by Maney and Goodson, 2011). During the breeding season, however, the message contained in song differs for male and female listeners. A female listening to male song is almost certainly being courted, whereas a male is being challenged by a territory holder or intruder. Song is therefore expected to have a more positive valence for females than for males. We predicted that neural responses to song in the females would resemble that of humans listening to liked music, whereas the pattern in the males would not.

The valence of song may be affected also by endocrine state. In *Zonotrichia* sparrows, females give a courtship display in response to song only when their plasma estradiol (E2) reaches breeding-typical levels (Moore, 1983; Maney et al., 2009). Males respond to song by singing back, and are more likely to do so if their testosterone (T) levels are elevated (Maney et al., 2009). Because the function of song, and behavioral responses to it, vary according to endocrine state, we manipulated plasma E2 in females and T in males in order to look at the effects on neural responses in the reward pathway. Following these manipulations, we exposed the birds to conspecific male song and quantified the expression of Egr-1 throughout the mesolimbic reward pathway. Because E2 treatment was expected to increase the valence of song, we predicted that responses would be greater in the E2-treated females than in untreated, non-breeding females. T-treatment was expected to lower the valence of an already negative stimulus, so we predicted little or no effect of T-treatment on the magnitude of mesolimbic reward responses in males.

## Materials and Methods

### Animals

All research was conducted in accordance with National Institutes of Health (NIH) principles of animal care, federal, and state laws, and university guidelines. Twenty-three white-throated sparrows of each sex were captured in mist nets during fall migration and housed initially in mixed-sex aviaries at the animal care facility at Emory University. The sex of the animals was confirmed *via* PCR analysis of a blood sample (Griffiths et al., 1998). Birds were housed under a short day length (10L:14D) for at least 4 months (Maney et al., 2007, 2008). The day length remained the same throughout the study to prevent gonadal recrudescence and elevation of endogenous E2 and T.

### Hormonal manipulation

Before the start of each experiment, birds were moved to individual cages (15″ × 15″ × 17″) inside walk-in sound-attenuating booths (Industrial Acoustics, Bronx, NY, USA). On the day of transfer, each bird received one subcutaneous silastic capsule (ID 1.47 mm, OD 1.96 mm, Dow Corning, Midland, MI, USA) sealed at both ends with A-100-S Type A medical adhesive (Factor 2, Lakeside, AZ, USA). Females received 12 mm capsules that were either empty (*n* = 11) or filled with 17β-estradiol (*n* = 12; Steraloids, Newport, RI, USA). Males received 15 mm capsules that were either empty (*n* = 11) or filled with T (*n* = 12; Steraloids). These doses elevate E2 and T to breeding-typical levels in this species (Maney et al., 2008, 2009; Sanford et al., 2010) and stimulated the E2-dependent courtship behavior known as copulation solicitation display (CSD) in this sample. After receiving the capsules, birds were housed in single-sex groups of 4–6 per booth for 7–9 days. All booths were identical.

### Stimulus presentation

On the afternoon prior to stimulus presentation, each bird was isolated by placing its cage inside an empty sound-attenuating booth equipped with microphone, speaker, and video camera. The stimulus playback began at 1 h after lights-on the following morning and was delivered *via* the speaker located inside the booth. The type of stimulus (song or tones, see below) was balanced across treatment groups for both males and females such that six hormone-treated and six blank-treated birds heard song, and six hormone-treated and five blank-treated birds heard tones. The stimuli were presented at a peak level of 70 dB measured at the bird’s cage (Maney et al., 2008). The stimulus presentation was followed by 18 min of silence. Video recordings of all birds were made during the stimulus presentation. For the females, we counted copulation solicitation events, defined as tail lifts, wing quivers, or vocalizations characteristic of CSD (see Maney et al., 2003). For the males, we counted full and partial songs (see Maney et al., 2009).

### Sound stimuli

#### Songs

White-throated sparrow songs obtained from the Borror Laboratory of Bioacoustics birdsong database were converted to AIFF format and background noise was removed. The recordings were edited so that a song was heard every 15 s, which mimics a natural song rate. Sequences of songs were then spliced together so that the identity of the singer changed to a novel male every 3 min. Presenting a variety of songs helps overcome habituation to the stimulus (Stripling et al., 1997). Each bird within a treatment condition (hormone or blank) heard 14 different singers, in a unique order determined by a balanced Latin square, for a total stimulus duration of 42 min.

#### Tones

For each of the 14 recordings of males singing, the frequency of each whistle (note) in one song was measured using AudioXplorer (Arizona Software, San Francisco, CA, USA). Songs usually contained five distinct frequencies. For each song, eight sinusoidal tones were generated at these frequencies and arranged in a random order 200 ms apart, resulting in a tone sequence that matched the song in duration, the average number of onsets and offsets, and total sound energy at each frequency. Tone sequences were spliced together as for the song stimuli, with 15 s of silence between each sequence, in an order determined by a balanced Latin Square.

### Histology

Sixty min following the onset of the stimulus presentation, birds were deeply anaesthetized with isoflurane (Abbott Laboratories, North Chicago, IL, USA) and decapitated. Ovaries were inspected to confirm a regressed state. Brains were harvested, fixed, and sectioned at 50 μm as previously described (Maney et al., 2003, 2007). Every third 50-μm section was incubated with an antibody against Egr-1 (cat# sc189; Santa Cruz Biotechnology, Santa Cruz, CA, USA), which was subsequently labeled using a biotinylated secondary antibody and avidin-biotin complex (Vector, Burlingame, CA, USA). The specificity of this antibody has been validated in this species *via* preadsorption studies (Saab et al., 2010). Labeling was visualized using diaminobenzidine enhanced with nickel (Maney et al., 2003, 2007). Sections were mounted onto gelatin-coated slides, dehydrated, and coverslipped in DPX (Sigma, St. Louis, MO, USA).

### Quantification of Egr-1 immunoreactivity

**Figure 1:**
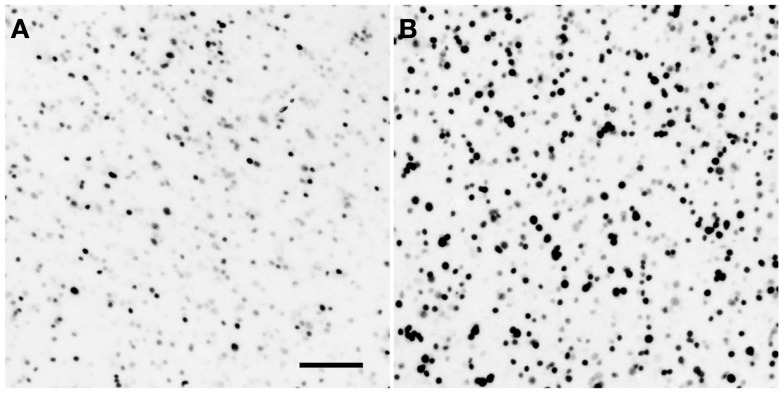
**Example of song-induced Egr-1 immunoreactivity**. Photomicrographs show the caudolateral nidopallium (putative homologue of the prefrontal cortex; see Table 1) in estradiol-treated female white-throated sparrows listening to synthetic tones **(A)** or conspecific song **(B)**. These examples depict the expression levels closest to each within-group median. Scale bar = 50 μm.

Examples of Egr-1 labeling are shown in Figure 1. We sampled from within the avian homologues of the nAc, caudate nucleus, Hp, medial amygdala, and VTA. We also sampled within an area proposed as an avian homologue of the prefrontal cortex and which receives a strong dopaminergic projection (Mogensen and Divac, 1982; Waldmann and Güntürkün, 1993). The names and abbreviations of each region of interest (ROI) and their human homologues are given in Table 1. Egr-1 immunoreactivity (ir) was quantified in six sections, 150 μm apart, in the VTA and in three sections in each of the other regions. Egr-1-ir was quantified in these regions on one side of the brain, chosen at random except when that region was damaged on one side due to folding or tearing of the section; in these cases the intact side was chosen. Images were acquired with a 4× (nAc and TnA) or 10× objective (all other regions) using a Leica DFC480 camera attached to a Zeiss Axioskop microscope. The light level on the microscope was set exactly the same for each picture.

**Table 1:** **Regions of the human mesolimbic reward pathway and their avian homologues**.

Human region	Avian region	Reference
Nucleus accumbens (nAc)	Nucleus accumbens (nAc)	Balint and Csillag (2007), Husband and Shimizu (2011)
Ventral tegmental area (VTA)	Ventral tegmental area (VTA)	Bottjer (1993), Reiner et al. (1994)
Caudate nucleus	Lateral striatum (LSt)	Karten (1969), Reiner et al. (1998)
Hippocampus (Hp)	Hippocampus (Hp)	Erichsen et al. (1991)
Medial amygdala (MeA)	Nucleus taeniae of the amygdala (TnA)	Cheng et al. (1999), Reiner and Karten (1985)
Prefrontal cortex (PFC)	Caudolateral nidopallium (NCL)	Mogensen and Divac (1982), Waldmann and Güntürkün (1993)

*The references cited are not comprehensive but rather represent examples illustrating homology between the human and avian brain regions*.

Egr-1-ir was quantified in each ROI by a blind observer using the thresholding feature in ImageJ (NIH). Briefly, the labeled nuclei with an optical density higher than a threshold value were counted within the entire ROI, obtained by tracing its borders in ImageJ (VTA) or within a circular area of approximately 0.2 mm^2^ placed within the ROI (all other regions). In most cases we used the threshold automatically set by ImageJ, which is based on contrast. Rarely, because of higher background staining, automatic thresholding caused obvious errors in the selection of labeled nuclei; in those cases the threshold was set manually. Our manual thresholding procedure has been shown to have high interrater reliability and low variability (Matragrano et al., 2011). The number of labeled nuclei per unit area was then calculated for each region by dividing the total number of nuclei counted by the total area sampled. A different set of data collected from the VTA, TnA, and Hp in the females was published elsewhere as part of a study on the social behavior network (Maney et al., 2008); in the current study, we quantified immunoreactivity in an alternate set of sections and the region sampled from TnA was smaller (three sections instead of eight).

### Statistical analysis

Data from males and females were analyzed separately. The values for cells per unit area in each region were square root transformed to normalize their distribution, then entered into a two-way MANOVA (SPSS) with treatment (hormone or blank) and stimulus (song or tones) as the independent variables. One missing data point for the VTA in the male dataset was generated in SPSS using the series mean. Significant main effects or interactions were followed by two-way ANOVAs for each region. When a main effect of stimulus or an interaction between stimulus and treatment was found, pairwise t-*tests* were conducted to compare across treatment within stimulus, and *vice versa*, for that region. When we found no interaction but *post hoc* tests showed an effect of stimulus in only one treatment group, we conducted power analysis (G*Power) to assess our ability to detect a significant effect in the other treatment group. To test whether the birds’ own behavior could explain Egr-1 expression, we used Spearman correlation tests to assess relationships between the Egr-1 expression in each region and CSD behavior (females) or singing (males).

## Results

### Females

**Figure 2:**
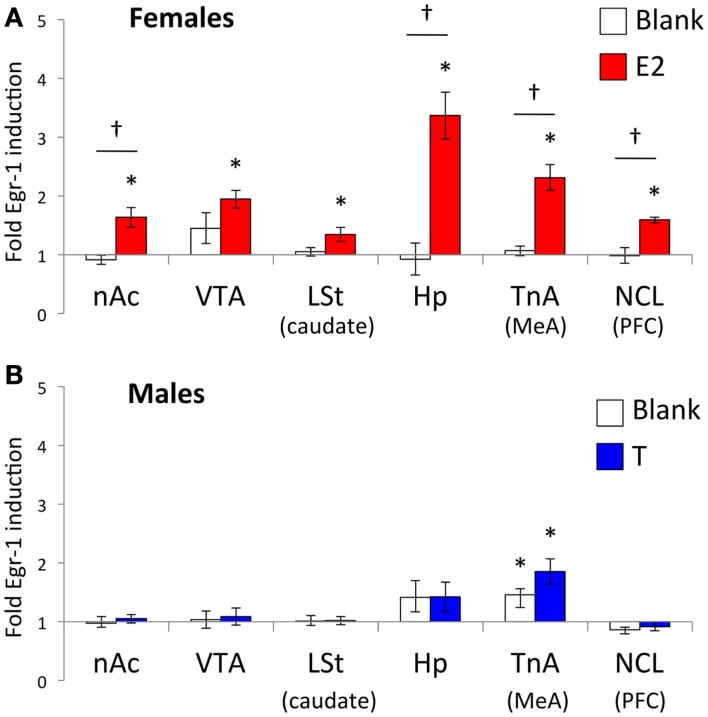
**Normalized Egr-1 responses in the mesolimbic reward system in (A) female and (B) male white-throated sparrows listening to conspecific male song**. For each region, cell density values were divided by the mean tone (control) value to create a normalized Egr-1 fold-induction scale (Jarvis et al., 1995). Values greater than 1 indicate that the mean response to song was greater than the mean response to tones for that region. In the E2-treated females, the response to song was significantly higher than the response to tones in each ROI measured. In the males, we observed selective Egr-1 responses only in TnA. *Significantly greater Egr-1 induction to song than tones; ^†^ Significant interaction between hormone treatment and auditory stimulus.

Egr-1 induction in the ROIs in females is plotted in Figure 2A, and the statistical analysis is summarized in Table 2. There were overall effects of both stimulus (*Wilks-lambda* *F*_6, 14_ = 3.348; *P* = 0.029) and treatment (*Wilks-lambda* *F*_6, 14_ = 4.494; *P* = 0.010), as well an interaction between the two (*Wilks-lambda* *F*_6, 14_ = 5.042; *P* = 0.006), indicating that E2 treatment modulated the neural response to auditory stimuli in these regions. *Post hoc* analyses showed that in the blank-treated birds, the Egr-1 response to song was not higher than that to tones in any ROI. In contrast, E2 treatment resulted in selective Egr-1 induction in all ROIs (Figure 2A). In all ROIs, the response to song was higher in E2-treated birds than in blank-treated birds.

**Table 2:** ***F*, *P*, and η^2^ for the effects of auditory stimulus and hormone treatment in females**.

	Effects of stimulus and treatment									Pairwise comparisons, *P*			
	Stimulus			Treatment			Stimulus × treatment			song vs. tones		E2 vs. blank	
Region	*F*_1, 22_	*P*	η^2^	*F*_1, 22_	*P*	η^2^	*F*_1, 22_	*P*	η^2^	E2	Blank	Song	Tones
nAc	4.855	**0.040**	0.130	2.894	0.105	0.078	9.652	**0.006**	0.259	**0.007**	0.467	**0.010**	0.302
VTA	12.313	**0.002**	0.311	6.099	**0.023**	0.154	2.043	0.169	0.052	**0.002**	0.232*	**0.022**	0.471
LSt (caudate)	4.801	**0.041**	0.117	14.222	**0.001**	0.347	2.743	0.114	0.067	**0.022**	0.712^†^	**0.008**	0.078
Hp	8.626	**0.008**	0.182	11.121	**0.003**	0.234	7.880	**0.011**	0.166	**<0.001**	0.943	**0.001**	0.751
TnA (MeA)	20.359	**<0.001**	0.363	0.023	0.880	<0.001	14.913	**0.001**	0.266	**<0.001**	0.602	**0.023**	**0.021**
NCL (PFC)	4.398	**0.039**	0.159	1.135	0.300	0.037	5.475	**0.030**	0.176	**0.001**	0.948	**0.019**	0.450

*The avian regions of interest are listed under “Region,” with human homologues in parenthesis when the nomenclature differs (see Table 1 for abbreviations). Significant effects in bold*.

*Power = 0.97.

*^†^Power = 0.75*.

In the females that performed CSD (E2-treated birds hearing song, *n* = 6), there was a significant negative correlation between CSD behavior and Egr-1 expression in the homologue of the medial amygdala, TnA (Spearman’s *rho* = −0.886; *P* = 0.019) and a trend in the LSt (Spearman’s *rho* = −0.771; *P* = 0.072). The negative correlation between behavior and the Egr-1 response in these regions, together with a lack of significant correlations in the other regions, helps to rule out the possibility that the Egr-1 response to song was caused by the birds’ behavioral responses to playback. In most cases the variability in CSD behavior did not explain the variability in Egr-1 expression. When it did, the direction of the relationship indicated that our main finding was not likely to be attributable to CSD behavior.

### Males

Egr-1 induction in the ROIs in males is plotted in Figure 2B, and the statistical analysis is summarized in Table 3. There were significant overall effects of stimulus (*Wilks-lambda* *F*_6, 14_ = 3.981; *P* = 0.016) and treatment (*Wilks-lambda* *F*_6, 14_ = 6.071; *P* = 0.003), but these effects did not interact with each other (*Wilks-lambda* *F*_6, 14_ = 0.241; *P* = 0.955). Two-way ANOVAS for each region revealed a significant effect of treatment in the VTA and Hp, and trends in the nAc, TnA (MeA), and NCL (BLA/PFC; see Table 3). In no case, however, did those effects interact with stimulus. Hearing song-induced a selective Egr-1 response only in TnA (MeA), and this response was not affected by endocrine state.

**Table 3:** ***F*, *P*, and η^2^ values for the effects of auditory stimulus and hormone treatment in males**.

	Effects of stimulus and treatment									Pairwise comparisons, *P*			
	Stimulus			Treatment			Stimulus × treatment			song vs. tones		E2 vs. blank	
Region	*F*_1, 22_	*P*	η^2^	*F*_1, 22_	*P*	η^2^	*F*_1, 22_	*P*	η^2^	E2	Blank	Song	Tones
nAc	0.015	0.904	0.001	3.445	0.079	0.151	0.220	0.645	0.010	–	–	–	–
VTA	0.158	0.696	0.006	8.528	**0.009**	0.307	0.066	0.800	0.002	–	–	–	–
LSt (caudate)	0.038	0.847	0.002	0.005	0.947	<0.001	0.016	0.900	0.001	–	–	–	–
Hp	1.784	0.197	0.067	5.577	**0.029**	0.210	0.315	0.581	0.012	–	–	–	–
TnA (MeA)	18.598	**<0.001**	0.440	3.275	0.086	0.077	1.381	0.254	0.033	**0.005**	**0.030**	0.052	0.678
NCL (PFC)	2.667	0.119	0.122	0.002	0.963	<0.001	0.154	0.700	0.007	–	–	–	–

**Post hoc* pairwise tests were performed only for regions for which the two-way ANOVAs showed a significant effect of stimulus or an interaction between stimulus and treatment. The avian regions of interest are listed under “Region,” with human homologues in parenthesis when the nomenclature differs (see Table 1 for abbreviations). Significant effects in bold*.

The number of songs given by the males during the stimulus presentation was correlated with Egr-1 expression only in the VTA (Spearman’s *rho* = 0.629; *P* = 0.001). When the groups were assessed individually, correlations between Egr-1 expression in the VTA and song production remained significant only in birds treated with T (song: Spearman’s *rho* = 0.928; *P* = 0.008; tones: Spearman’s *rho* = 0.986; *P *< 0.001). This result is consistent with other reports that IEG expression in the VTA is correlated with song rate (e.g., Maney and Ball, 2003). There were no significant correlations between song and Egr-1 expression in any other region.

## Discussion

### Song-induced Egr-1 responses in the mesolimbic reward pathway

In this study, we showed evidence of neural responses in the reward pathway of songbirds listening to conspecific song. These responses were significantly greater than those to behaviorally irrelevant control sounds in all of our regions of interest only in females with breeding-typical levels of E2. In non-breeding females treated with placebo, the response to song was not different from the response to control sounds. These results are consistent with studies in other species showing behavioral evidence of the incentive salience of song for receptive females (Eriksson and Wallin, 1986; Gentner and Hulse, 2000; Riebel, 2000).

In males, we found a main effect of T-treatment on Egr-1 expression in several regions of interest, which is consistent with other findings that gonadal steroids alone, independent of sensory stimuli, can induce IEG activity (Charlier et al., 2005; Sanford et al., 2010). We found an effect of stimulus only in the avian homologue of MeA, the TnA. The response in males was thus qualitatively quite different from the response in females and did not involve the nAc or VTA.

### Comparing neural responses in songbirds and humans

Our overall goal in this study was to compare the neural responses in the mesolimbic reward system of songbirds listening to conspecific song with those of humans listening to music. We compare below the pattern of Egr-1 responses observed in this study with the published literature describing BOLD (fMRI) and rCBF (PET) responses in humans listening to music. As a caveat, is important to note that BOLD and PET responses are qualitatively different both from each other and from IEG responses, and we should not assume that an increase in one signal always accompanies an increase in the other. BOLD and IEG signals do overlap extensively, however, when both techniques are applied in the same animals (Lazovic et al., 2005; Stark et al., 2006).

#### Nucleus accumbens and VTA

In the human literature, the brain region most commonly reported to respond to music is the ventral striatum, which includes the nAc. Responses in the human ventral striatum can be elicited by pleasant, liked, or happy music and are not elicited by unpleasant or sad music (Blood and Zatorre, 2001; Menon and Levitin, 2005; Koelsch et al., 2006; Mitterschiffthaler et al., 2007; Osuch et al., 2009; Montag et al., 2011; Pereira et al., 2011; Salimpoor et al., 2011). In the current study, E2-primed females showed more Egr-1 expression in both regions after hearing song than after hearing tones. Thus, at least in birds for whom song is expected to have a positive valence, the nAc does respond to song, as does its primary source of dopaminergic input, the VTA. Because activity in the VTA – nAc pathway is strongly associated with reward, this result is our best evidence hearing song may be a rewarding experience.

For males and non-breeding females, hearing song may be predictive of a fight. For females in breeding condition, however, hearing male song may precede rewarding activities such as courtship and copulation. We must therefore consider the possibility that song-induced Egr-1 responses in the reward pathway indicate the anticipation of reward, not reward *per se*. A number of authors have argued that activity in the nAc is better correlated with the expectation of a reward than actually receiving the reward (Schultz et al., 1992; Ikemoto and Panksepp, 1996; Knutson et al., 2001). Salimpoor et al. (2011) examined neural responses to music during an “anticipation” phase that occurs before the onset of chills, or during “peak emotional arousal,” defined by the onset of those chills. They found that anticipation was associated with BOLD responses in the caudate nucleus, not the nAc. In contrast, responses in the nAc were associated with peak emotional arousal. Their study suggests that under some conditions, in this case pleasurable auditory stimulation, neural responses in the nAc can be associated with the experience of reward. Here, we detected responses in both the nAc and the caudate (Figure 2A; Table 2). Most of the females in this study could not have associated male song with mating because at our collection site, 70% of the white-throated sparrows are hatch-year birds – meaning that although they are adults, they do not have breeding experience (DLM, unpublished observation). It is therefore unlikely that song-induced neural responses in the reward pathway were caused solely by a learned association between hearing song and engaging in sexual behavior. Because hatch-year females are certainly attracted to song regardless of their experience, however, we cannot rule out the possibility that nAc responses indicate appetitive reward or incentive salience rather than consummatory reward.

#### Caudate nucleus

Many investigators have reported caudate responses in humans listening to pleasurable music (Blood and Zatorre, 2001; Koelsch et al., 2006; Mitterschiffthaler et al., 2007; Berns et al., 2010; Montag et al., 2011; Salimpoor et al., 2011). Berns et al. (2010) reported that the intensity of BOLD responses in the caudate was strongly correlated with participants’ ratings of the likeability of a song. Here, we show that Egr-1 responses in the avian homologue of the caudate-putamen, the LSt, were greater to song than to control sound only in receptive females. Note that Salimpoor et al. (2011) reported BOLD responses in the caudate nucleus during the anticipation phase of music listening, but not during peak emotional responses. Wise (2004) argued that dopamine release in the dorsal striatum, which includes the caudate, is triggered less by the receipt of reward and functions more to establish and maintain the behaviors that bring about the reward.

#### Hippocampus

Of all the Egr-1 responses observed in this study, the largest was in the Hp. This region was previously shown to respond to conspecific song in zebra finches of both sexes (Bailey et al., 2002; Bailey and Wade, 2005). Notably, the BOLD signal in this region also increased in response to unpleasant, fearful, or sad music (Baumgartner et al., 2006; Koelsch et al., 2006; Mitterschiffthaler et al., 2007). Blood and Zatorre (2001) reported that in humans listening to their favorite music, the PET signal is actually reduced in the Hp. The region is heavily influenced by the emotional context of the stimulus (reviewed by Koelsch, 2010) and its precise role in reward needs further study.

#### Amygdala

Perhaps because it is heavily interconnected with the Hp, the response of the amygdala to music often mirrors the Hp response (Blood and Zatorre, 2001; Baumgartner et al., 2006; Koelsch et al., 2006; Eldar et al., 2007; Mitterschiffthaler et al., 2007; Pereira et al., 2011). These responses often depend on the valence of the stimulus, increasing with negative (e.g., fearful, sad) music and decreasing with pleasant music (Blood and Zatorre, 2001; Baumgartner et al., 2006; Koelsch et al., 2006; Mitterschiffthaler et al., 2007; Lerner et al., 2009). Blood and Zatorre reported that during listening to pleasurable music, rCBF in the amygdala was inversely related to that in the area containing the nAc. Interestingly, BOLD responses in the amygdala have also been reported to increase with familiar, pleasant, or even happy music (Brown et al., 2004; Leaver et al., 2009; Pereira et al., 2011). Ball et al. (2007), who observed amygdalar BOLD responses to both unpleasant and pleasant music alike, argued that the amygdala is involved in attributing either positive or negative valence to a stimulus.

Although the amygdala is a heterogeneous region containing several nuclei with distinct functions (reviewed by Swanson and Petrovich, 1998), it is usually treated in human imaging studies as a single entity (see Ball et al., 2007). Responses to emotionally arousing stimuli are perhaps most typical of the basolateral amygdala (see Ball et al., 2007), an area which has not been well-studied in songbirds (Martínez-García et al., 2008). In contrast, the avian amygdalar region homologous to MeA is better understood. In both mammals and birds, the region plays a critical role in a variety of social behaviors, particularly sexual behavior and aggression, by participating in the processing of sensory signals from conspecifics. In rats, for example, IEG expression is induced in the MeA by exposure to pheromones (Fiber et al., 1993; Bressler and Baum, 1996). In species representing three different avian orders, lesions of TnA may disrupt the processing of visual or auditory signals from conspecifics (Thompson et al., 1998; Cheng et al., 1999). Here, we found that hearing male song induces selective Egr-1 expression in TnA in males (Figure 2B) and E2-treated females (Figure 2A).

Although the MeA does appear to mediate the processing of emotional and possibly rewarding sensory stimuli, it seems unlikely that a response in this part of the pathway alone, as was seen here in T-treated males, indicates that the signal has incentive salience. Note that in humans, listening to sad or unpleasant music stimulates the amygdala and Hp without stimulating the nAc or caudate (Koelsch et al., 2006; Mitterschiffthaler et al., 2007). The pattern of human neural responses to negatively valenced music does not match perfectly what we found in males, in that the Hp responses we observed here were highly variable. Nonetheless, it is possible that a male sparrow’s experience of hearing song shares some qualities with the human experience of hearing unpleasant music. Overall, in order to compare responses in the amygdala with the response to music in humans, improved resolution to discern BOLD responses in the individual nuclei of the amygdala is needed.

#### Prefrontal cortex

The PFC responds in humans listening to music (Blood and Zatorre, 2001; Leaver et al., 2009; Lerner et al., 2009; Osuch et al., 2009), particularly music that evokes emotional memories (Janata, 2009). Although avian homologues of cortical regions are never obvious, the polymodal association region NCL has been proposed as the homologue of PFC (Mogensen and Divac, 1982). This area is the target of a dopaminergic projection that is clearly visible in tyrosine hydroxylase-stained tissue (Waldmann and Güntürkün, 1993; DLM, unpublished observation) and is understood to be part of a pathway involved in learned behaviors (Braun et al., 1999). In zebra finches, the area shows selective Egr-1 responses to conspecific song (Bottjer et al., 2010). We found, as was the case for the other regions of the reward pathway, that such induction occurs only in females treated with E2. In T-treated males and in blank-treated birds of both sexes, the response to song in NCL was indistinguishable from the response to behaviorally irrelevant tones.

### Regions outside the mesolimbic reward pathway that respond to music and song

Our main goal in this study was to compare the pattern of neural responses in songbirds listening to song with those of humans listening to music. In doing so, we focused on the mesolimbic reward pathway. There are, however, additional regions of the human brain that respond to music and which have homologues in birds. To complete our comparison, we summarize those findings below.

#### Hypothalamus

In humans listening to music, BOLD responses in the nAc and the VTA were strongly correlated with responses in the hypothalamus (Menon and Levitin, 2005), a region that is heavily interconnected with areas of the reward pathway, such as the VTA and MeA, in all vertebrates (reviewed by Goodson, 2005). Menon and Levitin argued that the hypothalamus is responsible for orchestrating the physiological responses, such as chills and changes in breathing and heart rate, that occur with music listening. The hypothalamus is also an important region in the songbird response to song. Song-induced IEG expression has been described in the medial preoptic area, mediobasal hypothalamus, and ventromedial hypothalamus in songbirds of both sexes (Goodson et al., 2005; Maney et al., 2007, 2008). The hypothalamus likely regulates physiological responses to song, which include reproductive hormone release (Wingfield and Wada, 1989; Maney et al., 2007) and heart rate (Dooling and Searcy, 1979). Ultimately, these physiological responses may contribute in important ways to the overall experiences of hearing song and music.

#### Periaqueductal gray

Although it is not traditionally considered part of the mesolimbic dopamine system, the PAG is interconnected with the VTA and plays an important role in opioid-mediated reward (reviewed by Le Merrer et al., 2009). In songbirds, opioids have been implicated in the motivation to sing (reviewed by Riters, 2011), but their role in rewarding listening is not well understood. Hearing conspecific male song induces Egr-1 expression in the PAG in E2-treated female white-throated sparrows (Maney et al., 2008). Likewise, Blood and Zatorre (2001) reported that listening to intensely pleasurable music induced PET responses in the area of the midbrain containing the PAG.

## Conclusion

Comparisons of music and song are usually limited to the bioacoustic analysis of the signals themselves. Here, we compared the responses of the receivers of the signal – the listeners. We found that song is similar to music in that they both induce responses in components of the mesolimbic reward pathway. In receptive females, every region of this pathway that has been reported to respond to music in humans, and that has a clear avian homologue, responded to song. In T-treated males, we detected responses only in the avian homologue of the MeA. This pattern, in other words a response in the amygdala without responses in the nAc or caudate, is typical of humans listening to unpleasant or fearful music (Baumgartner et al., 2006; Koelsch et al., 2006; Mitterschiffthaler et al., 2007). We cannot conclude that T-treated males find song unpleasant, however, because we see the same MeA response in females, and because amygdalar responses to emotionally arousing stimuli are more typical of the BLA than the MeA (reviewed by Fanselow and LeDoux, 1999). We can conclude only that overall, just as the message contained in song depends on the sex and endocrine state of the listener, so does the Egr-1 response.

At first, this result may seem to distinguish song from music, the pleasantness of which is not thought to vary along these parameters (cf. Panksepp, 1995; Sanders and Wenmoth, 1998). It is important to recognize, however, that in seasonally breeding animals, endocrine state provides context; reproductive hormones alter interpretations of and responses to social signals (reviewed by Maney, 2010). Responses to music are well-known to depend on context; for example, BOLD responses to fearful music can be strengthened or weakened by concurrent presentation of visual stimuli (Baumgartner et al., 2006; Eldar et al., 2007). Similarly, the likeability of music can be affected by the opinions of peers (Berns et al., 2010). It is thus not surprising that social context, in this case defined by endocrine state, affects the experience of hearing song.

Both song and music elicit responses not only in brain regions associated directly with reward, but also in interconnected regions that are thought to regulate emotion. The involvement of this circuit in music listening suggests that hearing music activates evolutionarily ancient neuroaffective mechanisms usually reserved for stimuli that, like song in birds, are critical for reproduction and survival. The adaptive value of bird song may be obvious; less obvious is the fact that music shares many of the same social functions. Like song, for example, music facilitates social contact, reduces conflict, communicates emotional state, and helps maintain interpersonal attachments (reviewed by Koelsch, 2010). These important, shared functions may underlie the evolution of emotional responses to both music and song. All humans know what it feels like to hear their favorite song or a discordant racket. The results of the present study suggest that to songbirds, hearing conspecific song may result in similarly emotional experiences.

The musical quality of bird songs has provided the impetus for many comparisons between the two sounds (e.g., Darwin, 1871; Dobson and Lemon, 1977; Catchpole and Slater, 1995; Marler, 2001; Miller, 2001; Slater, 2001; Baptista and Keister, 2005; Hartshorne, 2008; Araya-Salas, 2012). The current comparison provides evidence for shared neural responses. Some non-avian species, however, show similar behavioral and neural responses to sexually motivated vocalizations with far less aesthetic appeal to the human listener. In some frogs and toads, for example, females will approach playbacks of male advertisement calls (reviewed by Narins et al., 2007). In receptive female túngara frogs (*Engystomops pustulosus*), exposure to conspecific male calls induced Egr-1 expression in the ventral striatum (Chakraborty et al., 2010; cf. Hoke et al., 2010), suggesting that reward pathways may respond to courtship vocalizations in frogs as well as songbirds. Thus, although our findings may show that music and song induce similar responses in the listeners, these responses may be shared also by other animals listening to sounds that are much less musical. For that reason, reward or emotion in the listener is not a particularly convincing parallel between song and music when considered by itself. But song is like music in several other important ways. First, the temporal patterns and tonal qualities of bird song make it more complex and perhaps more technically challenging to perform than the croaks and bellows of many other animals. Second, the song of birds is learned and thus amenable to shaping by local culture and tastes (Baptista and Keister, 2005). It is possible that the complex musical structure, learned tradition, and pleasing nature of both song and music combine in a way that results in similar processing – most likely involving telencephalic structures that we did not examine here. Perhaps techniques will someday be developed to image neural responses in baleen whales, whose songs are both musical and learned (Tyack and Sayigh, 1997), and whose cortical neuroanatomy is more easily compared with humans (Hof and Van der Gucht, 2007).

## Conflict of Interest Statement

The authors declare that the research was conducted in the absence of any commercial or financial relationships that could be construed as a potential conflict of interest.
